# Supplementary material for: Correlation between Previous Antibiotic Exposure and COVID-19 Severity. A Population-Based Cohort Study
Source: Antibiotics (Basel). 2021 Nov 8;10(11):1364. doi: 10.3390/antibiotics10111364 (PMC8615228; doi:10.3390/antibiotics10111364)
Supplement: Supplementary file 1 [file antibiotics-10-01364-s001.zip › antibiotics-1396673-supplementary.pdf]

ONLINE SUPPLEMENTAL MATERIAL TABLES

TABLE S1. Family of antibiotic dispensed[*].

ATC classification	Family name and antibiotics	No (% of the total)
J01A	Tetracyclines	3,951 (1.0)
	- J01AA02 Doxycycline	3,340 (0.8)
	- Others	609 (0.2)
J01C	Penicillins	181,815 (47.9)
	- J01CA04 Amoxicillin	86,804 (22.9)
	- J01CF02 Cloxacillin	3,824 (1.0)
	- J01CE02 Penicillin V	2,315 (0.9)
	- J01CR02 Amoxicillin and clavulanic acid	87,512 (23.1)
	- Others	360 (0)
J01D	Other beta-lactams (cephalosporins)	21,871 (5.8)
	- J01DC02 Cefuroxime	274 (0.0)
	- J01DD08 Cefixime	5,249 (1.4)
	- J01DD16 Cefditoren	1,901 (0.5)
	- J01DB05 Cefadroxil	730 (0.2)
	- Others	717 (0.2)
J01E	Sulfonamides and trimethoprim	10,309 (2.7)
	- J01EE01 Cotrimoxazole	10,272 (2.7)
	- Others	37 (0)
J01F	Macrolides, lincosamides and streptogramins	63,783 (16.8)
	- J01FA01 Erythromycin	737 (0.2)
	- J01FA07 Josamycin	401 (0.1)
	- J01FA09 Clarithromycin	8,078 (2.1)
	- J01FA10 Azithromycin	47,802 (12.6)
	- J01FF01 Clindamycin	5,985 (1.6)
	- J01RA04 Spiramycin	1,046 (0.2)
	- Others	147 (0)
J01G	Aminoglycosides	192 (0.1)

J01M	Quinolones	54,317 (14.3)
	- J01MA02 Ciprofloxacin	23,130 (6.1)
	- J01MA06 Norfloxacin	972 (1.1)
	- J01MA12 Levofloxacin	25,306 (6.7)
	- J01MA14 Moxifloxacin	1,851 (0.5)
	- Others	58 (0)
J01X	Other antibacterials	43,284 (11.4)
	- J01XX01 Fosfomycin	4,430 (10.7)
	- J01XE01 Nitrofurantoin	2,243 (0.6)
	- Others	202 (0)
Total		379,522

ATC=Anatomical Therapeutic Chemical.

*Only antibiotics that account for at least 0.1% of the total are shown.

TABLE S2. Marginal structural model of the adjusted association between exposure to the most common families of antibacterial agents among patients with COVID-19 infection and the composite severity endpoint, combining death, hospitalization and/or pneumonia related to COVID-19 infection.

	Patients exposed to antibiotics and diagnosed with non-severe COVID-19 (n: 130,828)	Patients exposed to antibiotics and diagnosed with COVID-19 with the composite severity endpoint (n: 15,828)	Univariable		Multivariable*	
			OR (95% CI)	P-value	OR (95% CI)	P-value
J01C – Penicillins**	71,065 (54.3)	6,680 (42.2)	0.61 (0.59 - 0.63)	<0.0001	0.72 (0.69 -0.74)	<0.0001
J01D - Cephalosporins**	6,270 (4.8)	1,261 (8.0)	1.72 (1.61 - 1.83)	<0.0001	1.45 (1.36 - 1.55)	<0.0001
J01F - Macrolides, lincosamides and streptogramins**	23,985 (18.3)	2,708 (17.1)	0.92 (0.88 - 0.96)	0.0001	0.99 (0.94 - 1.04)	0.6446
J01M – Quinolones**	14,402 (11.0)	3,268 (20.6)	2.10 (2.02 - 2.19)	<0.0001	1.49 (1.43 - 1.56)	<0.0001
J01X - Other antibacterial agents**	12,448 (9.5)	1,357 (8.6)	0.89 (0.84 - 0.95)	0.0001	0.91 (0.85 - 0.96)	0.0017

*Weighted for age, sex, socioeconomic status and adjusted for the variables included in the table: smoking, body mass index, comorbidities of interest, concomitant medication. Final set of predictors selected by means of AIC-based stepwise algorithm.

**Compared to other individuals exposed to antibiotics

TABLE S3. Marginal structural models of the adjusted association between exposure to antibiotics among patients with COVID-19 infection and severe endpoints related to COVID-19.

Variable	No	Yes	Univariate		Multivariate*	
			OR (95% CI)	P-value	OR (95% CI)	P-value
Pneumonia						
No.	275,525	5,154				
Exposed to antibiotics	1435,81 (52.1)	3,075 (59.7)	1.33 (0.91 - 1.93)	0.1394	1.18 (0.81 - 1.74)	0.3877
High exposure (≥5 antibiotics)**	14,580 (5.3)	335 (6.5)	1.08 (0.97 - 1.21)	0.1794	0.95 (0.84 - 1.06)	0.3489
Recent exposure (<2 months)**	57,347 (39.9)	1,829 (59.5)	2.20 (2.05 - 2.37)	<0.0001	2.11 (1.96 - 2.28)	<0.0001
Exposed to HPCIAs**	46,132 (32.1)	1,345 (43.7)	1.64 (1.53 - 1.76)	<0.0001	1.58 (1.47 - 1.70)	<0.0001
Hospitalization						
No.	264,242	16,437				
Exposed to antibiotics	136,477 (51.6)	10,179 (61.9)	1.44 (1.29 - 1.60)	<0.0001	1.14 (1.02 - 1.27)	0.0222
High exposure (≥5 antibiotics)**	13,449 (5.1)	1,466 (8.9)	1.54 (1.45 - 1.63)	<0.0001	1.09 (1.02 - 1.16)	<0.01
Recent exposure (<2 months)**	53,725 (39.4)	5,451 (53.6)	1.78 (1.71 - 1.85)	<0.0001	1.55 (1.49 - 1.62)	<0.0001
Exposed to HPCIAs**	43,327 (31.7)	4,150 (40.8)	1.48 (1.42 - 1.54)	<0.0001	1.30 (1.25 - 1.36)	<0.0001
Death						
No.	272,704	7,975				
Exposed to antibiotics	141,402 (51.9)	5,254 (65.9)	1.37 (1.21 - 1.57)	<0.0001	1.10 (0.96 - 1.26)	0.1797

High exposure (≥5 antibiotics)**	13,754 (5.0)	1,161 (14.6)	2.63 (2.46 - 2.82)	<0.0001	1.44 (1.33 - 1.55)	<0.0001
Recent exposure (<2 months)**	56,999 (40.3)	2,177 (41.4)	1.05 (0.99 - 1.11)	0.1026	0.77 (0.72 - 0.82)	<0.0001
Exposed to HPCIAs**	45,263 (32.0)	2,214 (42.1)	1.55 (1.46 - 1.64)	<0.0001	1.18 (1.11 - 1.25)	<0.0001

HPCIA=Highest priority critically important antimicrobial.

*Weighted for age, sex, socioeconomic status and adjusted for the variables included in the table: smoking, body mass index, comorbidities of interest, concomitant medication. Final set of predictors selected by means of AIC-based stepwise algorithm.

**Compared to other individuals exposed to antibiotics.

TABLE S4. Codes related to COVID-19 infection considered (ICD-10 + thesaurus).

ICD-10 code	Description
B97.29	Other types of coronavirus diseases causing classified diseases elsewhere
B34.2	Non-specified coronavirus infection
B97.21	Coronavirus infection associated with severe acute respiratory syndrome (SARS) causing classified diseases elsewhere
J12.81	Coronavirus pneumonia associated with severe acute respiratory syndrome (SARS)
J12.89	Other types of viral pneumonia
Z20.828	Suspected COVID-19 infection

ICD-10=International Classification of Diseases, 10th revision
